# Conditional *ERK3* overexpression cooperates with *PTEN* deletion to promote lung adenocarcinoma formation in mice

**DOI:** 10.1002/1878-0261.13132

**Published:** 2021-12-14

**Authors:** Sreeram Vallabhaneni, Jian Liu, Marion Morel, Jixin Wang, Francesco J. DeMayo, Weiwen Long

**Affiliations:** ^1^ Department of Biochemistry and Molecular Biology Boonshoft School of Medicine Wright State University Dayton OH USA; ^2^ Zhejiang University‐University of Edinburgh Institute (ZJU‐UoE Institute) Zhejiang University School of Medicine Zhejiang University Haining China; ^3^ Hangzhou Cancer Institution Affiliated Hangzhou Cancer Hospital Zhejiang University School of Medicine Zhejiang University Hangzhou China; ^4^ Reproductive & Developmental Biology Laboratory National Institute of Environmental Health Sciences (NIEHS) Research Triangle Park NC USA

**Keywords:** ERBBs, ERK3, lung adenocarcinoma, NRG1, oncoprotein, PTEN

## Abstract

ERK3, officially known as mitogen‐activated protein kinase 6 (MAPK6), is a poorly studied mitogen‐activated protein kinase (MAPK). Recent studies have revealed the upregulation of *ERK3* expression in cancer and suggest an important role for ERK3 in promoting cancer cell growth and invasion in some cancers, in particular lung cancer. However, it is unknown whether ERK3 plays a role in spontaneous tumorigenesis *in vivo*. To determine the role of ERK3 in lung tumorigenesis, we created a conditional *ERK3* transgenic mouse line in which *ERK3* transgene expression is controlled by Cre recombinase. By crossing these transgenic mice with a mouse line harboring a lung tissue–specific Cre recombinase transgene driven by a club cell secretory protein gene promoter (CCSP‐iCre), we have found that conditional *ERK3* overexpression cooperates with phosphatase and tensin homolog (*PTEN*) deletion to induce the formation of lung adenocarcinomas (LUADs). Mechanistically, *ERK3* overexpression stimulates activating phosphorylations of erb‐b2 receptor tyrosine kinases 2 and 3 (ERBB2 and ERBB3) by upregulating Sp1 transcription factor (SP1)–mediated gene transcription of neuregulin 1 (*NRG1*), a potent ligand for ERBB2/ERBB3. Our study has revealed a bona fide tumor‐promoting role for ERK3 using genetically engineered mouse models. Together with previous findings showing the roles of ERK3 in cultured cells and in a xenograft lung tumor model, our findings corroborate that ERK3 acts as an oncoprotein in promoting LUAD development and progression.

## Graphical abstract

In this study, we generated a conditional *ERK3* transgenic mouse line and have found that conditional overexpression of *ERK3* in lungs cooperates with the deletion of the *PTEN* tumor suppressor to promote the formation of lung adenocarcinoma (LUAD). Our findings corroborate that ERK3 acts as an oncoprotein in promoting LUAD development and progression and is a potential therapeutic target for treating LUADs.

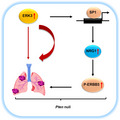

**Abbreviations**
CCSP: club cell secretory protein
ERBB2: erb‐b2 receptor tyrosine kinase 2
ERBB3: erb‐b2 receptor tyrosine kinases 3
ERK3: extracellular signal‐regulated kinase 3
IHC: immunohistochemistry
LUAD: lung adenocarcinoma
LUSC: lung squamous carcinoma
MAPK6: mitogen‐activated protein kinase 6
MK5: MAP kinase–activated protein kinase 5
MMP: matrix metalloproteinase
NRG1: neuregulin 1
NSCLC: non‐small cell lung cancers
PTEN: phosphatase and tensin homolog

## 1 Introduction

Extracellular signal–regulated kinase 3 (ERK3), also known as MAPK6, is a member of the atypical mitogen‐activated protein kinases (MAPKs) [1]. It is considered an atypical MAPK in that ERK3 signaling is not organized as classical three‐tiered kinase cascades, and its kinase domain harbors a Ser‐Glu‐Gly (SEG) activation motif instead of the Thr‐Xaa‐Tyr (TXY) activation motif shared by the classical MAPKs such as ERK1 and ERK2 [1, 2]. While still much less is known about the molecular actions of ERK3 signaling in cancers in comparison with the well‐studied ERK1/2 signaling, recent years have seen a considerable gain of our understanding of the roles of ERK3 in cancer development. On the one hand, ERK3 has been shown to promote cancer cell growth and migration in culture conditions and tumor growth and metastasis in xenograft mouse models of different human cancers, including lung cancer [3, 4, 5, 6], head and neck cancer [7], and breast cancer [8, 9, 10]. On the other hand, the inhibitory roles for ERK3 in tumor cell growth and/or migration have also been reported in several other types of cancers, including melanoma [11], non‐melanoma skin cancer [12], hepatocarcinoma [13], and intrahepatic cholangiocarcinoma [14]. Taken together, these studies suggest that ERK3 plays either tumor‐promoting or tumor‐suppressive roles depending on specific cancer type. Several different molecular mechanisms underlying the cancer‐promoting role of ERK3 have been proposed, such as activating SRC‐3/PEA3‐mediated matrix metalloproteinase (*MMP*) gene transcription in lung cancers and breast cancer [3] and c‐Jun/AP1‐mediated *IL‐8* expression in colon cancer cells and MDA‐MB231 breast cancer cells [9]. In addition, ERK3 promotes cancer cell growth and invasion in both kinase‐dependent [3, 6] and kinase‐independent mechanisms [5, 9]. On the contrary, it is largely unclear how ERK3 inhibits the growth and invasiveness of melanoma and hepatocarcinoma cells.

ERBBs are a family of structurally homologous receptor tyrosine kinases (RTKs), consisting of ERBB1 [also known as epidermal growth factor receptor (EGFR)], ERBB2, ERBB3, and ERBB4 [15]. ERBB RTKs are activated by a subset of growth factor ligands, including EGF and neuregulins (NRGs). Ligand binding induces receptor homo‐dimerization or hetero‐dimerization, followed by conformation change and kinase activation. ERBB2 has no known ligand binding, and ERBB3 lacks kinase activity, which makes them a preferable pair for heterodimerization in response to the binding of ERBB3 ligands, mainly NRGs such as NRG1 [16]. Activated ERBBs promote the activation of multiple downstream signaling pathways, primarily Ras/RAF/ERK1/2 and PI3K/Akt. ERBB RTK signaling is frequently upregulated in human cancers and plays critical roles in promoting tumor development and progression [15, 17].

Phosphatase and tensin homolog deleted on chromosome 10 (PTEN) is a dual lipid and protein phosphatase [18, 19]. PTEN is a potent inhibitor of PI3K/Akt signaling and acts as a tumor suppressor in multiple human cancers [20]. Like many other tumor suppressor genes, *PTEN* is frequently dysregulated in cancers by genetic mutations (loss of function) and other molecular mechanisms, including downregulation of gene transcription and posttranslational modifications, leading to downregulation or even loss of protein expression and function. The roles of PTEN in tumor progression and metastasis have been studied in mice with tissue‐specific deletion of *PTEN* [21]. For example, conditional deletion of *PTEN* in lung respiratory epithelial cells of bigenic mice containing both floxed *PTEN* alleles and a Cre recombinase transgene driven by a club cell secretory protein gene promoter (CCSP‐Cre) caused bronchiolar hyperplasia [22], implying that another molecular alteration is required for lung tumor development within the context of PTEN loss.

Although recent studies have revealed important roles for ERK3 in promoting lung cancer cell growth in cultured cells and tumor growth in xenograft mouse models, it is unknown whether or not ERK3 plays a role in spontaneous lung tumorigenesis. To determine the role of ERK3 overexpression in lung tumorigenesis, we created a conditional *ERK3* transgenic mouse line in which *ERK3* transgene expression is driven by the ubiquitous *CAGGS* promoter and is controlled by Cre recombinase due to a floxed transcription STOP cassette inserted between the promoter and *ERK3* transgene. By crossing with a CCSP‐Cre mouse line, we have found that while conditional *ERK3* overexpression alone did not cause a clear phenotype in lungs, *ERK3* overexpression cooperates with *PTEN* deletion to induce the formation of lung adenocarcinomas. Mechanistically, ERK3 overexpression stimulates activating phosphorylations of ERBB3 and ERBB2 by upregulating SP1‐mediated *NRG1* gene transcription.

## 2 Materials and methods

### 2.1 Animal study

Animal work was done in accordance with protocols (AUP 970 and AUP 1057) approved by the Animal Care and Use Committees of Wright State University. Mice were housed in the Laboratory Animal Research (LAR) facility of Wright State University in a pathogen‐free setting with 12‐h light/12‐h dark cycle, temperatures of 68–72°F (˜18–23 °C) and 45–55% humidity. Mice were monitored at daily base. Any animals suffering clinical disease were examined and treated by a veterinarian. All efforts were made to minimize pain, discomfort, and distress. Mice were euthanized/asphyxiated by CO2 exposure following current AVMA (The American Veterinary Medical Association) guidelines and LAR’s standard operating procedures. Mice (1 : 1 ratio of males and females) at variable ages were sacrificed by exsanguination under anesthesia with ketamine/xylazine mixture at a dose of 100 mg·kg^−1^ BW ketamine plus 10 mg·kg^−1^ BW xylazine (i.p.). Lungs, regional lymph nodes, and livers were harvested for analyzing primary lung tumor growth and potential metastasis.

### 2.2 Generation of conditional *ERK3* transgenic mouse (LSL‐*ERK3*)

Conditional *ERK3* transgenic mouse was generated by using an established approach and following the procedures as previously described [23]. To generate an embryonic stem cell targeting construct, first, human *ERK3* cDNA from pcDNA3‐ERK3 plasmid [3] was subcloned by *Sal I* site into the shuttle vector RfNLIII (generously provided by Ming‐Jer Tsai at Baylor College of Medicine, Houston, TX). Next, the fragment containing *ERK3* cDNA and two homologous sequences for recombination with the base vector was released by KpnI/NheI digestion from the shuttle vector. The released DNA fragment and the targeting base vector were then electroporated into SW102 bacteria, subsequently leading to the generation of the targeting construct (pCAGGS‐LSL‐huERK3) through homologous recombination–based insertion of *ERK3* into the targeting base vector upstream of the ubiquitous CAGGS promoter and downstream of a Lox‐Stop‐Lox (LSL) cassette. The targeting construct was verified by sequencing throughout the huERK3 cDNA and junction components.

The gene targeting in AB2.2 embryonic stem (ES) cells (mouse strain 129S5 background) and production of chimeras from those ES cells were performed by the genetically engineered mouse core at Baylor College of Medicine. Briefly, the targeting construct was linearized by PAC I digestion. The CAGGS‐LSL‐*ERK3* transgene in the linearized targeting construct was specifically integrated through homologous recombination into *Rosa 26* gene locus in AB2.2 ES cells. Chimeras were then produced using the correctly targeted ES clones. Chimeras were bred with C57BL/6 mice for the generation of the founder LSL‐*ERK3* transgenic mice. Germline transmission of the allele integrated with the LSL‐hu*ERK3* transgene was determined by mouse tail DNA PCR genotyping following the experimental conditions and procedures as described previously [23].

### 2.3 Functional validation of conditional *ERK3* transgene expression in LSL‐*ERK3* mouse

To validate the induction of *ERK3* transgene expression by Cre protein, the LSL‐*ERK3* mouse line was crossed with the *CAGG‐Cre‐ER*™ *mouse* line in which Cre expression is induced by tamoxifen treatment (JAX stock #004682) [24]. The littermates were genotyped by PCR for the expression of *ERK3* transgenes and Cre following the procedures as described previously [25]. The littermates including both males and females at the age of 5 weeks were administered with tamoxifen (75 mg·kg^−1^ body weight) once per day for a total of 5 consecutive days. The mice were sacrificed 3 days after the final injection, and lungs were harvested for RNA and protein extraction.

### 2.4 Generation of LSL‐*ERK3*/CCSP‐iCre, *PTEN* ^F/F^/CCSP‐iCre and LSL‐*ERK3/PTEN* ^F/F^/CCSP‐iCre for lung tumorigenesis study

CCSP‐iCre mouse [26] and floxed *PTEN* mouse (PTEN^F/F^) [27] were generated previously. LSL‐*ERK3* mouse was mated with CCSP‐iCre to generate LSL‐*ERK3*/CCSP‐iCre mouse. LSL‐*ERK3*/CCSP‐iCre was then mated with *PTEN* ^F/F^ to generate LSL‐*ERK3*/*PTEN* ^F/F^/CCSP‐iCre mouse. Mice (including both males and females) at different ages were sacrificed. Lungs were perfused using 1× PBS. The left lobe of lungs was then fixed by perfusion with 10% paraformaldehyde (PFA) for the use of histological analyses. The right lobes were frozen in liquid N2 and stored for later RNA or protein extraction.

### 2.5 PCR genotyping

PCR genotyping using mouse tail DNA was performed following the experimental conditions and procedures as described previously [23]. PCR primers are listed in Table 1. The PCR conditions were as follows: step 1: 15 s at 95 °C; step 2: 40 s at 95 °C for denaturation; step 3: 40 s at 56 °C for annealing; step 4: 90 s of 72 °C for elongation; step 5: repeating 33 cycles of steps 2–4; final step: hold at 4 °C until use.

**Table 1:** PCR Primers for mouse genotyping.

Genes	Primers (5′‐3′)	Amplicon size (base pairs)
LSL‐*ERK3*	Forward: GCAACGTGCTGGTTATTGTG Reverse: ATTAAGGGCCAGCTCATTCC	400
*Rosa26*	Forward: AAAGTCGCTCTGAGTTGTTAT Reverse: GGAGCGGGAGAAATGGATATG	600
Cre‐ER*™*	Forward: CTCTAGAGCCTCTGCTAACC Reverse: CCTGGCGATCCCTGAACATGTCC	400
CCSP‐iCre	Forward: TCTGATGAAGTCAGGAAGAACC Reverse: GAGATGTCCTTCACTCTGATTC	500
*PTEN*	Forward (wild type): GATACTAGTAAGATAAAAACCAGTAGT Reverse (shared): GTCACCCAGGCCTCTTGTCAAGT	400
	Forward (floxed allele): GCTTGATATCGAATT CCTGCACC	550

### 2.6 Histopathology and immunohistochemistry

Histopathological analysis of PFA‐fixed and paraffin‐embedded lung tissues was performed by hematoxylin and eosin (H/E) staining and immunohistological staining following the procedures described in our previous study [26]. Briefly, for H/E staining, lung tissue sections (5 µm thickness) were dewaxed three times in xylene for 10 min each. Next, tissues were rehydrated for 5 min in each of the gradient ethanol concentrations (100%, 95%, 70%, 50%), followed by 5 min in double distilled water. The tissue sections were stained with hematoxylin (Vector Laboratories #H‐3401, Burlingame, CA, USA) and eosin (Sigma Aldrich #SLBH6215V, Saint Louis, MO, USA). For immunohistological staining, tissue slides were dewaxed and dehydrated as mentioned above. Antigen retrieval was then performed by treating the sections using antigen unmasking agent (Vector Laboratories #H 3300) in an electric pressure cooker (Cuisinart‐Model #CPC‐600) for 15 min at a high‐pressure setting. Thereafter, endogenous peroxidase activity of the tissues was blocked by incubating the slides in 3% hydrogen peroxide in methanol for 10 min. Next, tissues were blocked in 5% normal goat serum (Vector Laboratories # s‐1000) or MOM blocking reagents (Vector Laboratories, Cat# MKB‐2213). Subsequently, sections were incubated with primary antibodies at 4 °C overnight followed with biotinylated HRP‐conjugated secondary antibody (Vector Laboratories # BA‐1000) at room temperature for 1 h. The slides were then developed using Vectastain ABC kit (Vector Laboratories # PK‐6100) and diaminobenzidine (DAB, ACROS ORGANICS #112090250, Carlsbad, CA, USA) substrate reagent (freshly prepared 1.7 mm DAB in 50 mm Tris (pH 7.6) containing 0.05% hydrogen peroxide) and then counter stained with hematoxylin. The primary antibodies used for immunohistochemistry are anti‐ERK3 (1 : 50 dilution, Abcam #ab53277, Waltham, MA, USA), anti‐TTF1 (1 : 1000 dilution, DAKO #M3575, Santa Clara, CA, USA), anti‐P63 (1 : 50 dilution, Santa Cruz, # sc‐8431, Dallas, TX, USA), anti‐Ki67 (1 : 5000, Abcam #ab15580), anti‐cleaved caspase 3 (1 : 100; Cell Signaling Technology #CST9661, Danvers, MA, USA), and anti‐phospho‐ERBB3 (1 : 100, Cell Signaling Technology # CST4791).

### 2.7 Western blotting

Proteins were extracted from tissues, followed by Western blotting analysis following the procedures described previously [26]. Briefly, tissues were homogenized in EBC lysis buffer containing 1 mm Complete protease inhibitors cocktail (Roche Diagnostics, Indianapolis, IN, USA) and 1 mm protein phosphatase Inhibitor Cocktail I (Sigma Aldrich). Protein lysates were mixed with 5X SDS sample buffer and boiled then resolved on SDS‐PAGE gels, followed by transfer onto nitrocellulose membrane and Western blotting. The Western blot was visualized by ECL chemiluminescence (Thermo Scientific). The primary antibodies used are anti‐ERK3 (Abcam #ab53277), anti‐phospho‐ERK3 (S189) (generated by our own laboratory, [5]), anti‐MK5 (Sigma, #HPA015515), anti‐phospho‐MK5 (T182) (Abbexa, #abx11808, Cambridge, UK), anti‐Ki67 (Abcam #ab15580), anti‐PARP (Cell Signaling Technology, cat# 9532), anti‐EGFR (Santa Cruz, SC‐03), anti‐phospho‐EGFR (Cell Signaling, CST3777), anti‐ERBB2 (Santa Cruz, SC‐284), anti‐phospho‐ERBB2 (Santa Cruz, SC‐293110), anti‐ERBB3 (Santa Cruz, SC‐285), anti‐phospho‐ERBB3 (Cell Signaling, CST4791), anti‐Akt (Cell Signbaling, CST4691), anti‐phospho‐Akt (Cell signaling, CST4060), anti‐mTOR (Cell Signaling, CST2983), anti‐phospho‐mTOR (Cell signaling, CST2971), anti‐ERK1/2 (Cell signaling, CST4695), anti‐phospho‐ERK1/2 (Cell Signaling, CST4370), and anti–β‐actin (Sigma). β‐actin was used as a loading control in Western blotting analysis.

### 2.8 RNA extraction and real‐time quantitative PCR (RT‐qPCR)

Total RNA was extracted from cells using Trizol reagent (Thermo Scientific), and reverse transcription (RT) was done using SuperScript VILO Master Mix (Thermo Scientific) according to the manufacturer’s protocol. Quantitative PCR (qPCR) was performed using TaqMan Probe system (Roche Diagnostics) on the Applied Biosystems 7500 (Applied Biosystems) with either 18s RNA (for tissues) or GAPDH (for cells) as the internal control. Relative expression to normalizer sample was calculated using the ΔΔ*C*_T_ method.

### 2.9 Cell culture and siRNA transient transfection

H1299 lung cancer cells stably expressing a shRNA specifically against ERK3 (shERK3) or a non‐targeting control shRNA (shCtrl) were generated previously [3]. The H520 lung cancer cell line and HeLa cervical cancer cell line were obtained from ATCC. H1299 and H520 were maintained in RPMI 1640 medium supplemented with 10% fetal bovine serum (FBS). HeLa cells were cultured in Dulbecco’s modified Eagle medium (DMEM) supplemented with 10% FBS. All the culture media and supplements were purchased from Life Technologies/Invitrogen (Carlsbad, CA, USA). Transient transfection with siRNAs (20 nm working concentration) in H520 cells were done using DharmaFECT Transfection Reagent (Dharmacon, Lafayette, CO, USA) by following the manufacturer’s instructions. The silencer select siRNA targeting human ERK3 and the Silencer non‐targeting Control #1 were purchased from Ambion (Austin, TX, USA).

### 2.10 Luciferase reporter assay

pLightSwitch‐NRG1 promoter‐Luc (purchased from SwitchGear Genomics, Carlsbad, CA, USA) is a luciferase expressing construct containing the human NRG1 gene promoter (927‐bp fragment upstream of transcription start site). Plasmids pSG5‐ERK3, pSG5‐SP1 and the empty vector pSG5 were described in the previous study [28]. HeLa cells were co‐transfected with pLightSwitch‐NRG1 promoter‐Luc, pSG5‐ERK3, pSG5‐SP1, or pSG5‐empty vector control using lipofectamine 3000 Reagent (Invitrogen). The luciferase activity was measured 36 h post‐transfection using LightSwitch Luciferase Assay Kit (SwitchGear Genomics). To test the effect of PI3K inhibition on NRG1gene promoter activity, 24 h after plasmid transfection, HeLa cells were treated with Wortmanin (100 nm) or vehicle DMSO for 20 h, followed by cell lysis and luciferase activity measurement.

### 2.11 Statistics

Data are expressed as mean ± standard deviation (SD). Statistical significance was determined by one‐way analysis of variance (ANOVA) or two‐tailed Student’s *t* test. A *P*‐value of < 0.05 was considered statistically significant.

## 3 Results

### 3.1 Conditional *ERK3* overexpression and *PTEN* deletion induces tumorigenesis in mouse lungs

**Fig. 1:**
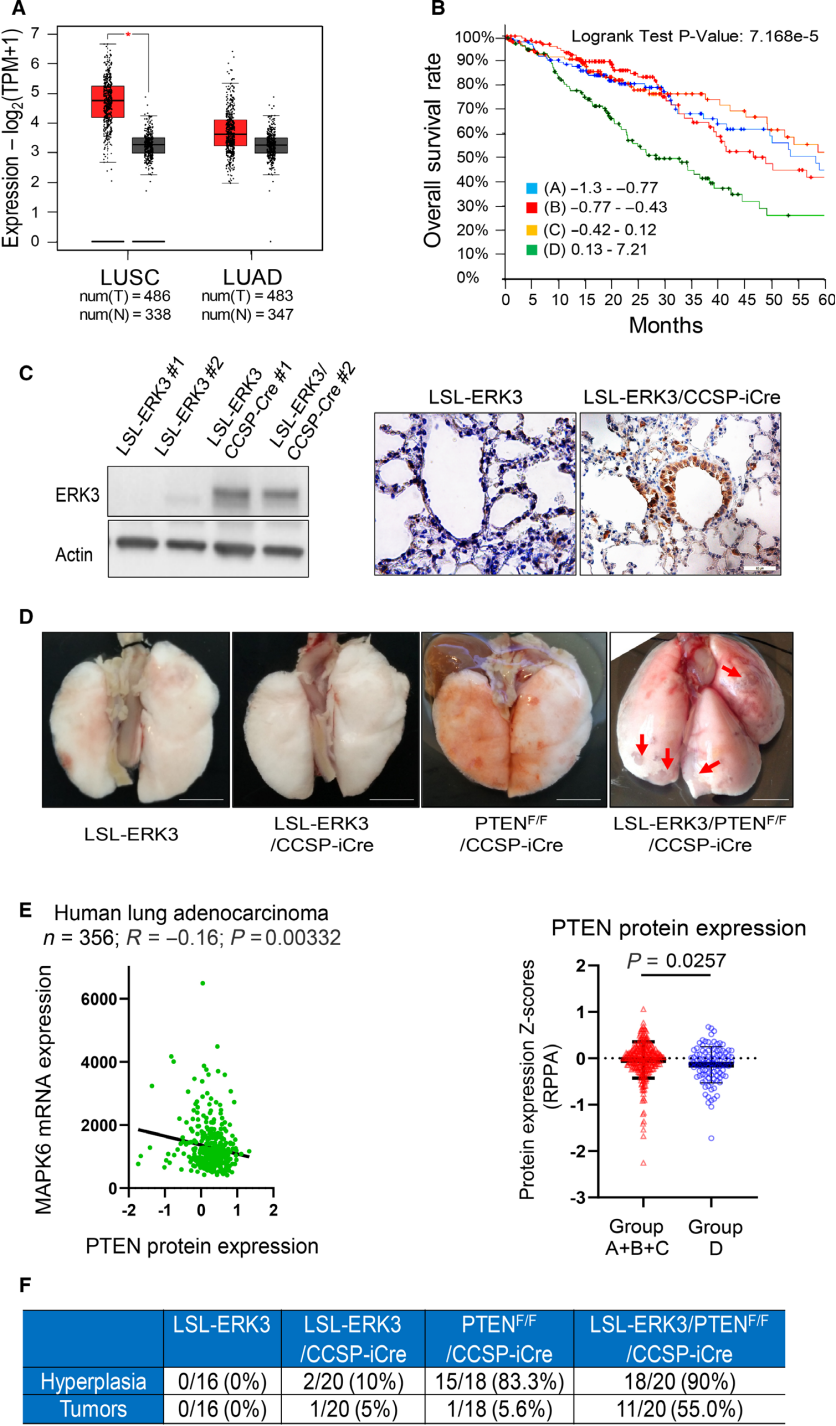
*ERK3* overexpression induced lung tumor growth in *PTEN*‐null background. (A) *ERK3* (*MAPK6*) gene expression is upregulated in NSCLCs. Differential *ERK3* mRNA expression in lung squamous carcinomas (LUSCs) or lung adenocarcinomas (LUADs) (from TCGA project) versus normal lung tissues (from both TCGA and GTEx projects) was performed using GEIPA2 web server. *P < 0.01, One‐way ANOVA. num: indicates the total number of samples. T: indicates tumor. N: indicates normal tissues. (B) Lung adenocarcinoma patients (*n* = 505, Data source: cBioportal/TCGA Lung adenocarcinoma Firehose legacy) were divided into four groups from quartiles of *MAPK6* (*ERK3*) mRNA expression (*Z* scores relative to diploid samples, RNASeq V2 RSEM): group A (*n* = 126; 42 decreased cases and median overall survival time: 58.41 months); group B (*n* = 127; 41 decreased cases and median overall survival time: 46.68 months); group C (*n* = 125; 36 decreased cases and median overall survival time: 66.59 months); group D (*n* = 127; 64 decreased cases and median overall survival time: 28.38 months). (C) ERK3 overexpression was induced in lungs by CCSP‐iCre. LSL‐*ERK3* mouse was crossed with CCSP‐iCre mouse to generate bigenic LSL‐*ERK3*/CCSP‐iCre mice. Cre‐mediated overexpression of ERK3 protein in lungs of 1‐year old bigenic LSL‐*ERK3*/CCSP‐iCre mice was first analyzed by Western blotting analysis (on the left) and then confirmed by immunohistochemical staining of ERK3 (images on the right). Scale bar = 100 µm. (D) Representative lungs of LSL‐ERK3 mice (*n* = 16), LSL‐*ERK3*/CCSP‐iCre mice (*n* = 17), *PTEN* ^F/F^/CCSP‐iCre mice (*n* = 15) and LSL‐*ERK3*/*PTEN* ^F/F^/CCSP‐iCre mice (*n* = 11) at the age of one and a half years. Scale bar = 0.5 cm. As compared to LSL‐*ERK3* mice and LSL‐*ERK3*/CCSP‐iCre mice, which had normal lungs, *PTEN* ^F/F^/CCSP‐iCre mice showed enlarged size of the lung and LSL‐*ERK3/PTEN* ^F/F^/CCSP‐iCre mice had tumors on the surface of the lung (indicated by arrows). (E) Among LUADs patients described in (B), 356 had data of PTEN protein expression [*Z* scores by Reverse phase protein assay (RPPA)]. PTEN protein expression levels in these patients are negatively correlated with *MAPK6/ERK3* mRNA transcript levels (Left panel). In addition, cohort D patients had significant lower PTEN protein level than that of patients of other cohorts (Right panel). Data are expressed as mean ± SD. Statistical test: two‐tailed Student’s *t* test. (F) Incidence of hyperplasia and formation of tumors in lungs of mice. The denominators and the numerators indicate the total number of mice analyzed in each group and the number of mice with lung hyperplasia or tumor formation, respectively.

Previous studies from our lab and others’ have shown that *ERK3* expression is upregulated in both lung adenocarcinomas (LUADs) and lung squamous cell carcinomas (LUSCs) of non–small‐cell lung cancers (NSCLC) and that ERK3 promotes lung cancer cell growth and invasiveness [3, 6]. One common limitation of previous analyses on *ERK3* expression in NSCLC is the limited number of normal lung tissues in comparison with that of tumor samples. Hence, we performed an analysis of *ERK3* mRNA expression in NSCLCs (either LUADs or LUSCs) utilizing the GEPIA2 web server that analyzes differential gene expression in tumors versus a large number of normal samples from both the TCGA and GTEx projects [29]. As shown in Fig. 1A, this analysis confirmed that *ERK3* mRNA expression was upregulated in both LUSC and LUAD. In addition, by analyzing the c‐Bioportal/TCGA NSCLC datasets [30], we found that high *ERK3* expression level indicates poor overall survival of patients with lung adenocarcinomas (LUADs) (Fig. 1B). These results suggest that *ERK3* overexpression may promote NSCLC growth and progression. To test this, first we generated a conditional human *ERK3* transgenic mouse line (LSL‐*ERK3*) in which the CAGGS‐LSL‐huERK3 transgene (Fig. S1A) was inserted specifically into *Rosa26* gene locus. To validate the functionality of the transgene, the LSL‐*ERK3* mouse line was crossed with the Cre‐ER*™* line in which Cre expression is induced by tamoxifen treatment [24]. The littermates were genotyped by PCR for the expression of Cre and LSL‐*ERK3* transgenes (Fig. S1B). Mice were then treated with tamoxifen for 5 days. As shown in Fig. S1C, tamoxifen‐induced *ERK3* transgene expression in lungs of bigenic LSL‐*ERK3*/*Cre‐ER™* mice.

**Fig. 2:**
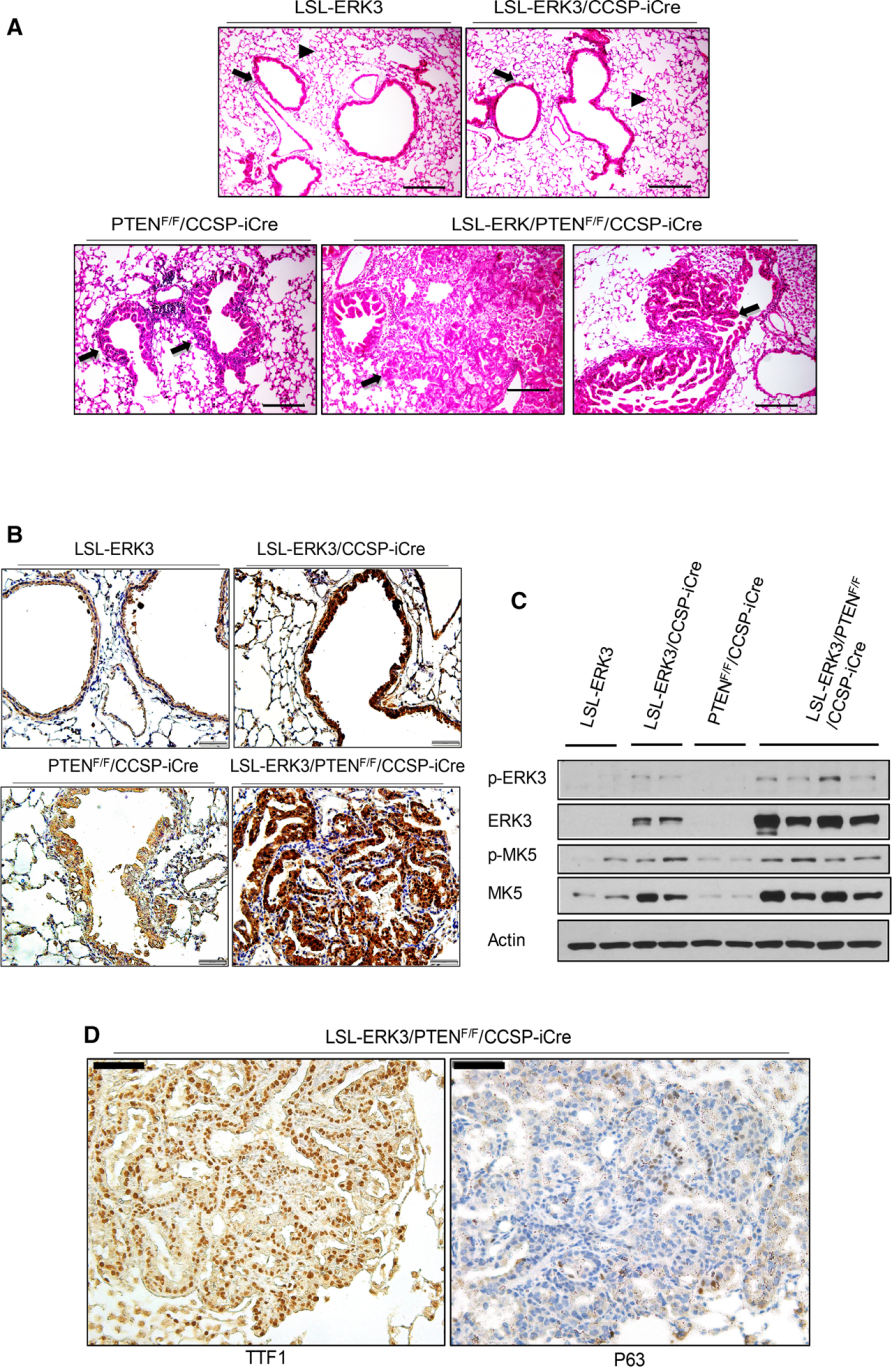
*ERK3* overexpression induced lung adenocarcinoma development in *PTEN*‐null background. (A) Representative hematoxylin and eosin (H/E) staining of formalin fixed and paraffin embedded (FFPE) lung sections of LSL‐*ERK3* (*n* = 16) and LSL‐*ERK3*/CCSP‐iCre mice (*n* = 17), both of which display normal terminal bronchioles (indicated by arrows) and surrounding alveoli (indicated by arrow heads), *PTEN* ^F/F^/CCSP‐iCre (*n* = 15) displaying hyperplasia (indicated by arrows) of the bronchiole epithelium, and LSL‐*ERK3/PTEN* ^F/F^/CCSP‐iCre mice (*n* = 11) displaying hyperplasia and tumors (indicated by arrows). Scale bar = 100 µm. (B) Representative IHC of ERK3 protein expression in lungs of LSL‐*ERK3* (*n* = 16), LSL‐*ERK3*/CCSP‐iCre mice (*n* = 17), *PTEN* ^F/F^/CCSP‐iCre (*n* = 15) and LSL‐*ERK3/PTEN* ^F/F^/CCSP‐iCre mice (*n* = 11). Scale bar = 50 µm. (C) Western blotting analysis of ERK3 phosphorylation at S189 (p‐ERK3), total ERK3 protein, MK5 phosphorylation at T182 (p‐MK5) and total MK5 protein levels in lungs of mice. (D) Representative IHC of TTF1 (a marker for LUAD) and P63 (a marker for LUSC) in the lungs of LSL‐*ERK3/PTEN* ^F/F^/CCSP‐iCre (*n* = 11). Scale bar = 100 µm.

Having successfully generated the conditional *ERK3* transgenic mouse line, we then crossed LSL‐*ERK3* mouse line with lung‐specific CCSP‐iCre line that expresses an improved Cre (iCre) inserted into the *CCSP* gene locus [26] to determine whether overexpression of *ERK3* in the lung causes spontaneous lung tumor formation (Fig. S1E). While ERK3 protein overexpression was demonstrated by both Western blotting and immunohistochemistry in the lungs of LSL‐*ERK3*/CCSP‐iCre mice (Fig. 1C), no apparent phenotype was observed in the lungs of these mice (compare LSL‐*ERK3*/CCSP‐iCre with LSL‐*ERK3* control mice in Figs 1D and 2A), indicating CCSP‐iCre–induced *ERK3* overexpression alone is insufficient for spontaneous lung tumorigenesis. Lung tumor formation usually requires multiple genetic alterations of both oncogenes and tumor suppressor genes [31]. *PTEN*, a tumor suppressor of the PI3K/Akt signaling pathway, is often downregulated by both genomic and non‐genomic mechanisms, leading to frequent loss of protein expression and function in human lung cancer [32]. In line with this, conditional *PTEN* deletion in lungs induced lung hyperplasia [22] and cooperated with oncogenic *KRas* to promote lung tumor growth [33]. Interestingly, we found that *ERK3* mRNA expression level was negatively correlated with PTEN expression level in LUADs (TCGA dataset, Fig. 1E left panel), and cohort D patients having the highest ERK3 expression level (Fig. 1B) had a significantly lower PTEN protein expression level than that of patients of other cohorts (right panel of Fig. 1E). As such, we attempted to investigate the role of *ERK3* overexpression in lung tumorigenesis under *PTEN* deletion background. For this purpose, we generated triple transgenic mice that harbor LSL‐*ERK3* transgene, *PTEN* floxed alleles (*PTEN* ^F/F^), and CCSP‐iCre transgene (LSL‐*ERK3*/*PTEN* ^F/F^/CCSP‐iCre, such as mouse #410 in Fig. S1D). As reported previously [22], CCSP‐iCre–mediated *PTEN* depletion induced lung hyperplasia but not tumor formation (CCSP‐iCre/*PTEN* ^F/F^, Fig. 1D). Importantly, tumors were observed on the surface of the lungs of LSL‐*ERK3*/*PTEN* ^F/F^/CCSP‐iCre mice (Fig. 1D), and tumor incidence was about 50% (Fig. 1F). These results demonstrate that conditional *ERK3* overexpression cooperates with *PTEN* deletion to induce lung tumorigenesis.

### 3.2 Concurrent *ERK3* overexpression and *PTEN* deletion induce the formation of lung adenocarcinoma

There are two major subtypes of NSCLC: LUAD and LUSC on the basis of the pathological morphology and expression of biomarkers [31, 33]. To know which subtype(s) of lung tumors are formed in LSL‐*ERK3*/*PTEN* ^F/F^/CCSP‐iCre mice, we first performed histological analysis by H/E staining. As shown in Fig. 2A, tumors appear to be acinar adenocarcinoma histologically. ERK3 overexpression in lung tumors was demonstrated by both immunohistochemistry (IHC) (Fig. 2B) and Western blotting analysis (Fig. 2C). In addition, ERK3 overexpression led to increase in activating S189 phosphorylation of ERK3 and T182 phosphorylation of MAP kinase–activated protein kinase 5 (MK5, a substrate of ERK3 [1]) (Fig. 2C), indicating overexpressed ERK3 is catalytically active. We then confirmed the LUAD formation by IHC of biomarkers. Indeed, tumors in LSL‐*ERK3*/*PTEN* ^F/F^/CCSP‐iCre mice show prominent expression of TTF1 (a biomarker of LUAD) and faint staining of p63, a biomarker of LSCC (Fig. 2D and Fig. S2). These results suggest that concurrent *ERK3* overexpression and *PTEN* deletion induce the formation of lung adenocarcinoma.

### 3.3 *ERK3* overexpression increases cell proliferation and reduces cell apoptosis in *PTEN*‐null background

**Fig. 3:**
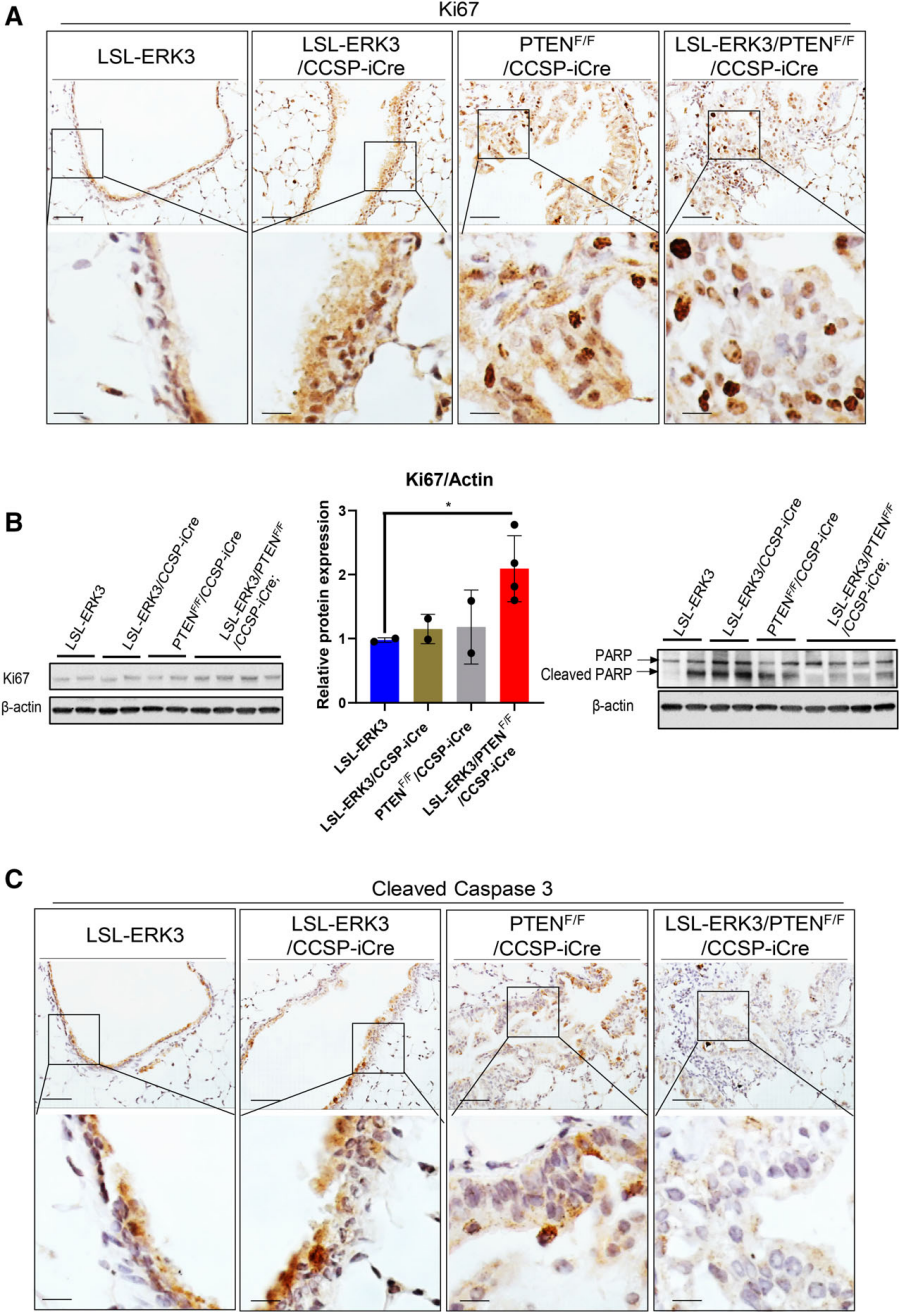
ERK3 overexpression increases cell proliferation and reduces cell apoptosis in *PTEN*‐null background. (A) Representative IHC of Ki67 protein expression in lungs of LSL‐*ERK3* (*n* = 11), LSL‐*ERK3*/CCSP‐iCre mice (*n* = 11), *PTEN* ^F/F^/CCSP‐iCre (*n* = 11), and LSL‐*ERK3/PTEN* ^F/F^/CCSP‐iCre mice (*n* = 11). Scale bar equals to 50 µm in the upper panels and 12.5 µm in the lower panels. (B) Western blot analyses of Ki67 and cleaved PARP levels in lungs of mice. The middle panel shows the quantification of Ki67 protein expression level (normalized by β‐Actin level) relative to that of LSL‐*ERK3* control (arbitrarily set as 1). Results are expressed as mean ± SD. * indicates *P* < 0.05 by two‐tailed Student’s *t* test. (C) Representative IHC of cleaved caspase 3 in lungs of LSL‐*ERK3* (*n* = 11), LSL‐*ERK3*/CCSP‐iCre mice (*n* = 11), *PTEN* ^F/F^/CCSP‐iCre (*n* = 11) and LSL‐*ERK3/PTEN* ^F/F^/CCSP‐iCre mice (*n* = 11). Scale bar equals to 50 µm in the upper panels and 12.5 µm in the lower panels.

Next, we examined the effects of *ERK3* overexpression on cell proliferation and survival in lungs. While conditional *ERK3* overexpression alone did not show a clear effect on expression levels of Ki67 (a cell proliferation marker) in lungs (compare LSL‐*ERK3*/CCSP‐iCre with LSL‐*ERK3* control mice in Fig. 3A,B), *ERK3* overexpression in the context of *PTEN* deletion greatly increased Ki67 expression level (compare LSL‐*ERK3*/*PTEN* ^F/F^/CCSP‐iCre with other groups in Fig. 3A,B). These results suggest that *ERK3* overexpression stimulates cell proliferation in *PTEN* deletion background. We then determined the effects on apoptosis by analyzing PARP cleavage and caspase 3 cleavage [34]. The levels of both cleaved PARP (Fig. 3B right panel) and cleaved caspase 3 (Fig. 3C) were greatly decreased in the lungs of LSL‐*ERK3*/*PTEN* ^F/F^/CCSP‐iCre compared with other groups, suggesting concurrent *ERK3* overexpression and *PTEN* deletion inhibits cell apoptosis. An increase in cell proliferation and a decrease in cell apoptosis account for the lung tumor formation in LSL‐*ERK3*/*PTEN* ^F/F^/CCSP‐iCre mice.

### 3.4 *ERK3* overexpression increases activating phosphorylation of ERBB2 and ERBB3 in the context of *PTEN* deletion

**Fig. 4:**
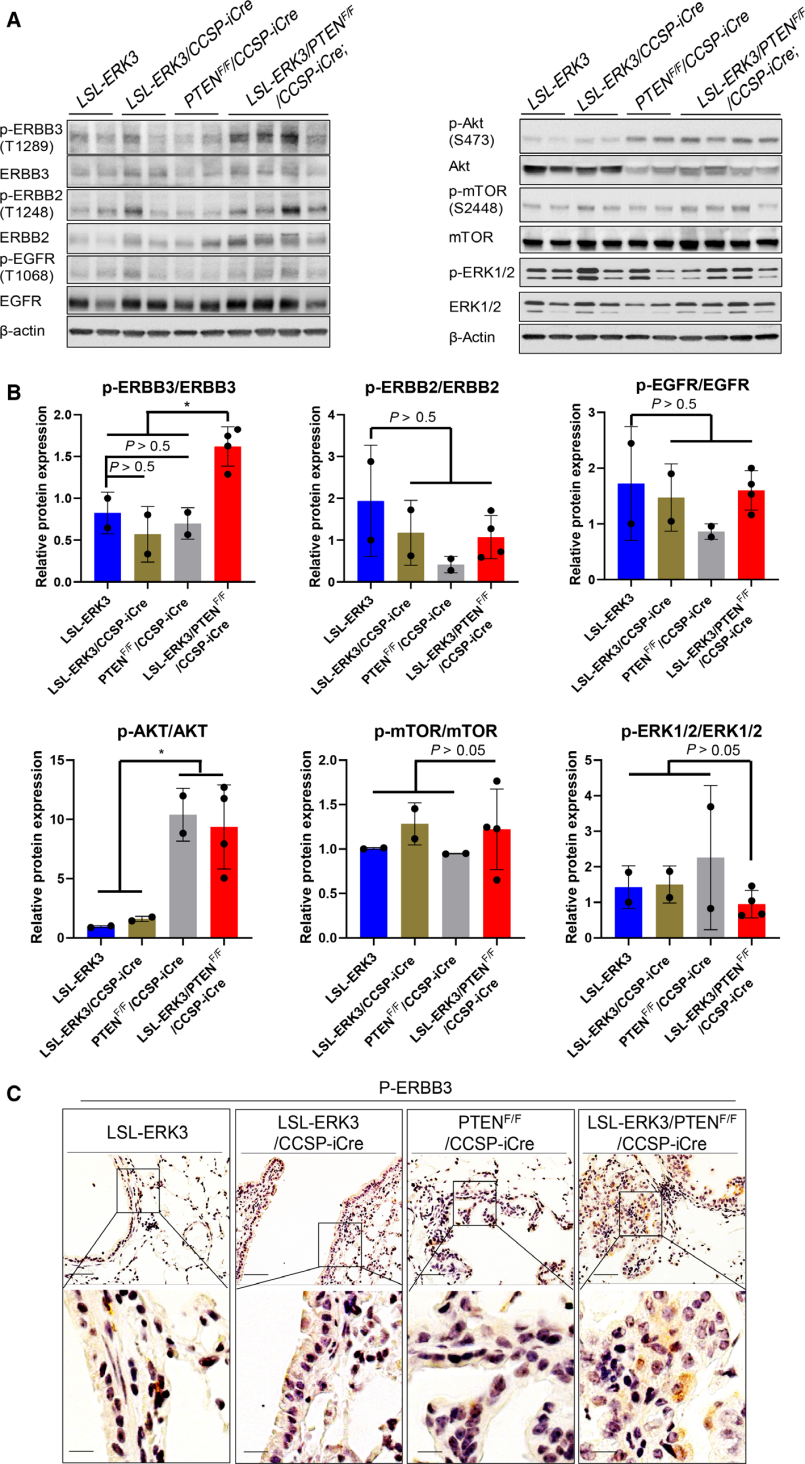
ERK3 overexpression increases the phosphorylation level of ERBB3 in *PTEN*‐null background. (A) Western blot analyses of activating phosphorylations of ERBBs (p‐ERBBs) and total ERBB protein levels (Left panel) and activating phosphorylations and total protein levels of Akt, mTOR and ERK1/2 in lungs of mice (Right panel). (B) Quantification of protein phosphorylation level (normalized by total protein level) of western blots shown in (A). Results are expressed as mean ± SD. *indicates *P* < 0.05 by one way ANOVA. (C) Representative IHC of p‐ERBB3 in lungs of mice LSL‐*ERK3* (*n* = 6), LSL‐*ERK3*/CCSP‐iCre mice (*n* = 6), *PTEN* ^F/F^/CCSP‐iCre (*n* = 6) and LSL‐*ERK3/PTEN* ^F/F^/CCSP‐iCre mice (*n* = 6). Scale bar equals to 50 µm in the upper panels and 12.5 µm in the lower panels.

ERBBs, in particular ERBB1, ERBB2, and ERBB3, play important roles in promoting lung tumor development and progression [35]. In an attempt to elucidate the molecular mechanism (s) by which *ERK3* overexpression promotes tumor development, we examined the effects of *ERK3* overexpression on the activating phosphorylations of ERBB1, ERBB2, and ERBB3. Interestingly, *ERK3* overexpression in the context of *PTEN* deletion in LSL‐*ERK3*/*PTEN* ^F/F^/CCSP‐iCre mice greatly increased the levels of activating phosphorylations of ERBB3 and ERBB2, whereas it had little effect on ERBB1 (EGFR) phosphorylation (Fig. 4A,B). As expected, Akt phosphorylation was greatly increased in *PTEN* ^F/F^/CCSP‐Cre mice (Right panel of Fig. 4A,B), but not further increased by ERK3 overexpression (compare LSL‐*ERK3*/*PTEN* ^F/F^/CCSP‐iCre with *PTEN* ^F/F^/CCSP‐iCre mice, Fig. 4A). Increase in ERBB3 phosphorylation in LSL‐*ERK3*/*PTEN* ^F/F^/CCSP‐iCre was confirmed by immunostaining of phospho‐ERBB3 in lungs (indicated by prominent cytoplasmic brown staining, Fig. 4C).

### 3.5 ERK3 upregulates *NRG1* gene transcript level in lung tumor cells

**Fig. 5:**
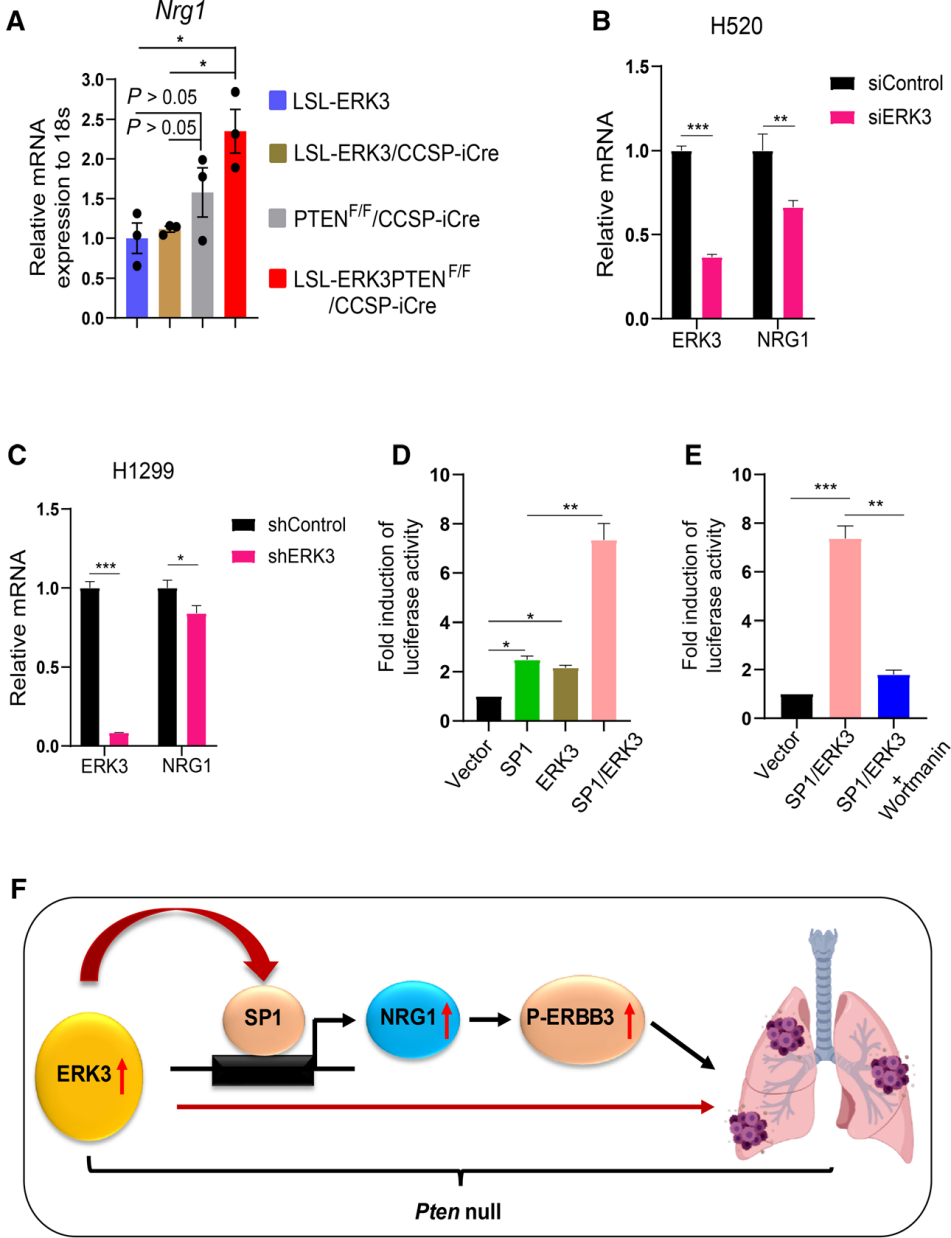
ERK3 upregulates the *NRG1* gene transcript level. (A) RT‐qPCR analysis of *NRG1* mRNA expression in lungs of mice. The *NRG1* mRNA expression level in mouse lungs of each different genotype was normalized to that of 18S and presented in relative to that of control LSL‐*ERK3* mouse lungs (arbitrarily set as 1). Results are expressed as mean ± SD of six mice of each different genotype. *indicates *P* < 0.05 by one‐way ANOVA. (B) RT‐qPCR analysis of *NRG1* mRNA expression levels in H520 lung cancer cells treated with a siRNA specifically against *ERK3* (siERK3) or a non‐targeting control siRNA (siCtrl). Values represent mean ± SD of three independent experiments. Statistical significance was determined by one‐way ANOVA. **P < 0.01, ***P < 0.001. (C) RT‐qPCR analysis of *NRG1* mRNA expression levels in H1299 lung cancer cells stably expressing a shRNA specifically against ERK3 (shERK3) or a non‐targeting control shRNA (shCtrl). Values represent mean ± SD of three independent experiments. *P < 0.05, ***P < 0.001. (D) ERK3 stimulates SP1‐mediated *NRG1* gene promoter activity. HeLa cells were co‐transfected with pLightSwitch‐NRG1 promoter‐Luc and ERK3, SP1 or pSG5‐empty vector control as indicated. The luciferase activity was measured 36 h post‐transfection using LightSwitch Luciferase Assay Kit (SwitchGear Genomics). Values in bar graphs present the fold induction of luciferase activity relative to the vector control. Results are expressed as mean ± SD of three independent experiments. *indicates *P* < 0.05 and **P < 0.01 (one‐way ANOVA). (E) Wortmanin treatment (100 nm for 20 h) greatly reduced *NRG1* gene promoter activity stimulated by ERK3/SP1. Plasmid transfection and luciferase assay were performed as described in (D). Results are expressed as mean ± SD of three independent experiments. **P < 0.01 and ***P < 0.001 (one way ANOVA). (F) A schematic diagram of lung adenocarcinoma formation induced by ERK3/SP1/NRG1/ERBB3 pathway in PTEN‐null background.

NRG1 is a major ligand for ERBB3 and induces heterodimerization and subsequent activation of ERBB3 and ERBB2 [16]. In addition, *NRG1* was shown to be upregulated in NSCLC and stimulate NSCLC growth [36, 37]. Thus, we determined whether ERK3 overexpression affected *NRG1* expression level, which may account for its effect in stimulating ERBB3/ERBB2 phosphorylation levels. Indeed, there is a significant increase in *NRG1* transcript level in lung tumor tissues of LSL‐*ERK3*/*PTEN* ^F/F^/CCSP‐iCre mice as compared to *PTEN* ^F/F^/CCSP‐iCre mice (Fig. 5A), suggesting that *ERK3* overexpression upregulates *NRG1* gene expression in the context of *PTEN* deletion. The upregulation of *NRG1* by ERK3 was confirmed in human lung cancer cell lines H520 and H1229, in which knockdown of ERK3 led to a significant decrease in *NRG1* transcript levels (Fig. 5B,C). The SP1 transcription factor is known to bind to the *NRG1* gene promoter and regulate its transcription [38, 39]. In addition, ERK3 was shown to stimulate SP1‐mediated *VEGFR2* gene transcription [28]. We therefore performed *NRG1* gene promoter–driven luciferase assay for testing whether ERK3 coactivates SP1‐mediated *NRG1* gene transcription. Indeed, co‐expression of SP1 and ERK3 synergistically stimulates *NRG1* gene promoter activity in driving luciferase gene expression (Fig. 5D). Given that PI3K/Akt pathway is highly activated upon *PTEN* deletion in tumors of LSL‐*ERK3*/*PTEN* ^F/F^/CCSP‐iCre mice, we examined the effect of PI3K inhibition on the activity of the *NRG1* promoter. Treatment with the PI3K inhibitor wortmannin greatly reduced *NRG1* gene promoter activity stimulated by ERK3/SP1 (Fig. 5E), suggesting that both ERK3 activity and the PI3K/Akt activity are important for the upregulation of NRG1 signaling.

## 4 Discussion

In recent years, accumulating studies have suggested an important role for ERK3 in promoting tumor cell growth and invasion in several cancers, including lung cancer. However, the role of ERK3 in spontaneous tumor growth in animal models has not been reported. In the present study, we have found that conditional overexpression of *ERK3* in lungs cooperates with *PTEN* deletion to promote the formation of lung adenocarcinoma at least partially owing to upregulation of NRG1/ERBB3 signaling (Fig. 5F). To our knowledge, our study is the first revealing a bona fide tumor‐promoting role for ERK3 *in vivo* using genetically engineered mouse models. Together with previous findings showing important roles of ERK3 in cultured cells and in the xenograft lung tumor model [3, 6], our findings corroborate that ERK3 acts as an oncoprotein in promoting LUAD development and progression.

*ERK3* mutations, including those in the kinase domain, have been reported in several types of cancer, but the frequency of these mutations is low [40, 41]. More frequent in cancers is the upregulation of *ERK3* expression. Several studies, including those in TCGA, have shown the upregulation of *ERK3* at both mRNA level and protein level in NSCLC, including both LUAD and LUSC [3, 6]. The kinase activity and cellular functions of ERK3 are positively regulated by phosphorylation of S189 in the activation motif, although it remains elusive regarding the upstream signal for stimulating S189 phosphorylation [5, 42, 43, 44]. Importantly, the level of S189 phosphorylation, which was determined by mass spectrometry–based phosphoproteomic analyses in the study, was shown to be significantly elevated in LUADs [6]. These clinic findings suggest that altered ERK3 signaling in cancers is mainly caused by upregulation of expression level and posttranslational modifications rather than genetic mutations.

ERK3 plays differential roles in cell growth in different types of cancers. In NSCLCs, the role of ERK3 on cell growth appears to be affected by other molecular alterations in cells. For example, while ERK3 depletion had little effect on the growth of lung cancer cell lines H1299 and H1650 that express wild‐type *KRAS* [3, 6], it greatly reduced cell growth and/or anchorage‐independent colony formation of *KRAS* ^G12C^‐positive H23 and H2122 NSCLC cell lines and xenograft tumor growth of the Calu‐1 cell line also expressing *KRAS* ^G12C^ [6]. Similarly, in our present *in vivo* transgenic mouse study, we found that *ERK3* overexpression alone did not show an apparent effect on lung epithelial cell growth (cell proliferation and apoptosis data). However, in the context of deletion of the *PTEN* tumor suppressor, *ERK3* overexpression increased cell proliferation, decreased cell apoptosis, and promoted tumor formation. These findings suggest that ERK3 itself may not be able to transform normal epithelial cells, but is capable of promoting cancer cell growth and invasiveness once cells are transformed following the loss‐of‐function mutation of tumor suppressor gene or gain‐of‐function mutation(s) of oncogenes.

In contrast with the well‐studied ERK1/2 signaling, little is known about the upstream stimuli and activators and downstream targets of ERK3. In an attempt to elucidate how ERK3 overexpression stimulates cell growth and tumorigenesis, we examined activating phosphorylation levels of ERBBs, ERK1/2, and Akt/mTOR, all of which are well‐known oncogenic pathways in NSCLCs [35, 45]. Importantly, we found that conditional *ERK3* overexpression in *PTEN* deletion background in lungs greatly increased the phosphorylation levels of ERBB3 and ERBB2 by upregulating their ligand *NRG1* gene transcript level. Significant upregulation of *NRG1* gene transcript was not seen in either *ERK3* overexpression alone or *PTEN* deletion alone (Fig. 5A), suggesting that both ERK3 signaling and Akt signaling are required for stimulating *NRG1* gene transcription in the lung epithelium, which is likely mediated by SP1. *NRG1* is a known target gene of the SP1 transcription factor [38, 39]. Akt phosphorylates SP1, thereby stimulating SP1 transcriptional activity [46, 47]. In addition, SP1 transcriptional activity is also regulated by its coactivators such as SRC‐3 [48, 49]. We have reported in our previous study that ERK3 phosphorylates SRC‐3, which stimulates the interaction of SRC‐3 with SP1 and their transcriptional activity in *VEGFR2* gene transcription [28]. Similarly in the present study, we found ERK3 greatly increased SP1 transcriptional activity on *NRG1* gene promoter. A major downstream target of NRG1/ERBB3/ERBB2 signaling is PI3K/Akt. However, we did not observe a clear concomitant increase of Akt phosphorylation with NRG1/ERBB3/ERBB2 activation in lungs of LSL‐*ERK3*/*PTEN* ^F/F^/CCSP‐iCre mice, likely in that Akt is constitutively phosphorylated/activated due to *PTEN* deletion.

Lung cancer can be originated from different cell types, such as type I and type II epithelial cells in the distal lung and club cells in the proximal airway [50]. Accordingly, cell type–specific lung tumor models have been generated by utilizing either club cell‐specific CCSP‐Cre‐ or type II epithelial cell–specific *SPC* (surfactant protein C)‐Cre–mediated expression of oncogenes (e.g. *Kras*) or deletion of tumor suppressors (e.g. *Trp53* and *Pten*) [51]. Although ERK3 is expressed in both epithelial cells and club cells [52], the cell‐autonomous functions of ERK3 in each of these cell types are unknown. Hence, the functions of ERK3 in lung tumor development and progression can be cell origin dependent. Therefore, it would be important to further investigate the roles of ERK3 in lung tumor development and progression using other Cre‐expressing systems such as using the SPC‐Cre mouse line or by intratracheal administration of Cre‐expressing adenoviruses into the lungs for targeting multiple cell types [53].

## 5 Conclusions

In summary, our study shows that conditional *ERK3* overexpression cooperates with *PTEN* deletion to promote the formation of lung adenocarcinoma at least partially by upregulating NRG1/ERBB3 signaling. Our findings corroborate that ERK3 acts as an oncoprotein in promoting LUAD development and progression and is a potential therapeutic target for treating LUADs.

## Conflict of interest

The authors declare no conflict of interest.

## Author contributions

The experiments were designed by WL and JL. SV, JL, MM, JW, and WL carried out the experiments and data analysis. The CCSP‐iCre mouse line was generated by FD. The manuscript was written by WL and JL with inputs and comments from all coauthors.

## Supporting information


**Fig. S1**. Generation of a transgenic mouse line conditionally expressing human ERK3.
**Fig. S2**. IHC staining of P63 in lung tumors and normal lung epithelium of LSL‐ERK3/PTEN^F/F^/CCSP‐iCre mouse.

## Data Availability

The data that support the findings of this study are included in Figs 1, 2, 3, 4, 5 and the Figs S1 and S2 of this article and are available from the corresponding authors upon reasonable request.
