# Supplementary material for: Conditional ERK3 overexpression cooperates with PTEN deletion to promote lung adenocarcinoma formation in mice
Source: Mol Oncol. 2021 Dec 14;16(5):1184–99. doi: 10.1002/1878-0261.13132 (PMC8895443; doi:10.1002/1878-0261.13132)
Supplement: Supplementary file 1 — Fig. S1. Generation of a transgenic mouse line conditionally expressing human ERK3. Fig. S2. IHC staining of P63 in lung tumors and normal lung epithelium of LSL‐ERK3/PTENF/F/CCSP‐iCre mouse. [file MOL2-16-1184-s001.docx]

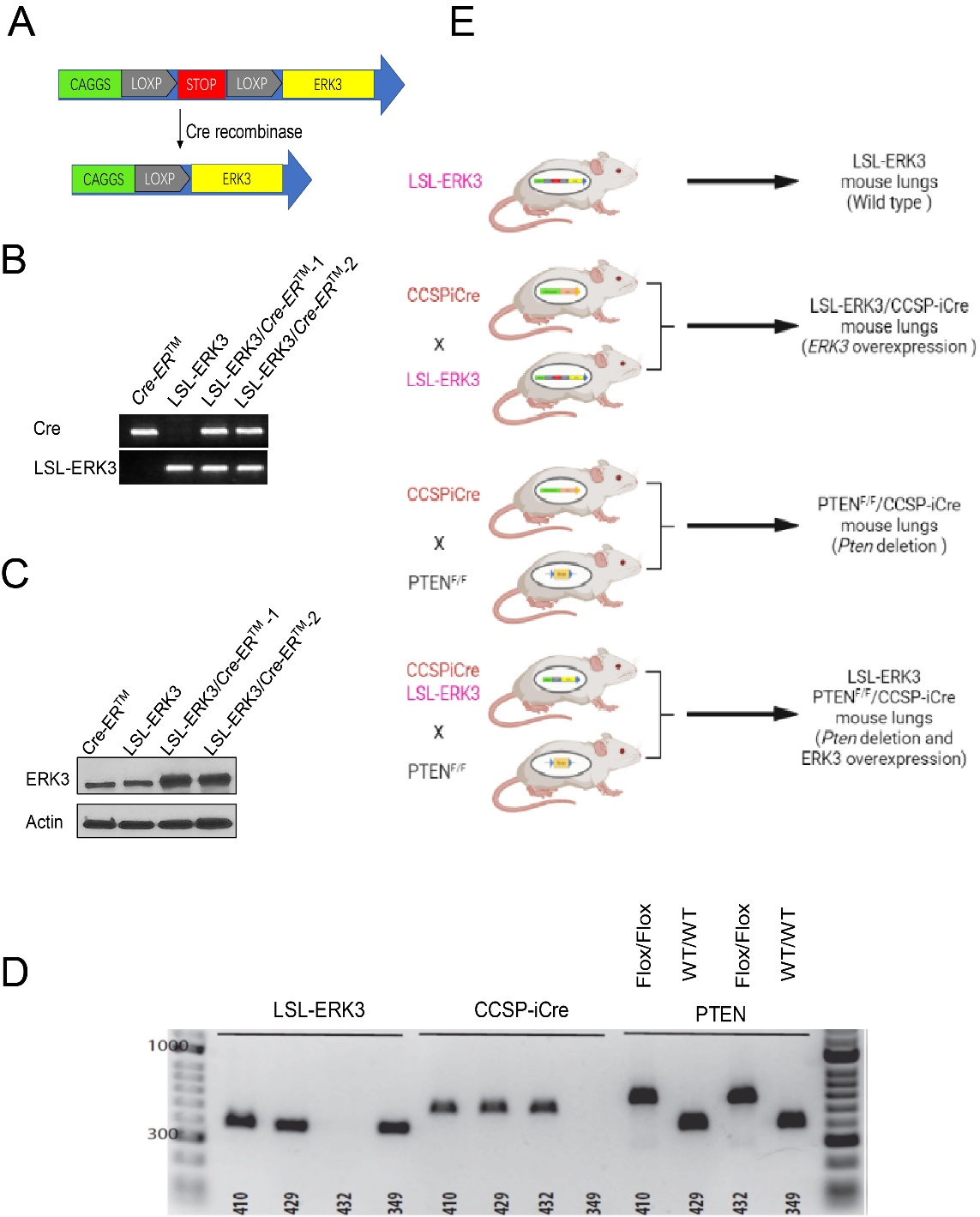


**Supplemental Figure 1 (Figure S1) Generation of a transgenic mouse line conditionally expressing human ERK3 (A)** Schematic illustration of conditionally controlled *ERK3* transgene expression by Cre recombinase. Human *ERK3* cDNA was cloned downstream of a ubiquitous CAGGS promoter, but was interspaced by a transcription STOP sequence that is flanked by two Lox P sites. The STOP sequence prevents the transcription of *ERK3* driven by CAGGS promoter. In the presence of Cre recombinase, the STOP sequence flanked by Lox P sites will be excised and ERK3 expression will then be activated. The transgenic mouse line is designated as LSL-*ERK3*. **(B and C)** Characterization of LSL-*ERK3* transgenic mouse line. LSL-*ERK3* mouse was crossed with the CAGG-Cre-ER^TM^ mouse line. The littermates were genotyped by PCR of tail DNA (**B**) for the presence of LSL-*ERK3* and *Cre* transgenes. Mice at the age of 5 weeks were administered with tamoxifen (75 mg/Kg body weight) once per day for a total of 5 consecutive days to induce *Cre* expression and the subsequent *ERK3* transgene expression. The mice were sacrificed 3 days after the final injection, and tissues were harvested for RNA and protein extraction. Western blot analyses of ERK3 protein expression in the lungs of different transgenic mice were shown in (**C**). IB: Immunoblot. (**D)** Representative PCR genotyping of experimental transgenic mice analyzed using tail DNA and specific primers for LSL-*ERK3* (amplicon size: 400bp), CCSP-iCre (amplicon size: 450 bp), *PTEN* wild type allele (amplicon size: 400bp) and floxed *PTEN* allele (amplicon size- 450 bp). mouse #349: LSL-*ERK3*; mouse#432: *PTEN*^F/F^/CCSP-iCre; Mouse #429: LSL-*ERK3*/CCSP-iCre; mouse #410: LSL-*ERK3/PTEN*^F/F^/CCSP-iCre. (**E**) A schematic diagram showing the production of transgenic mice in the study.


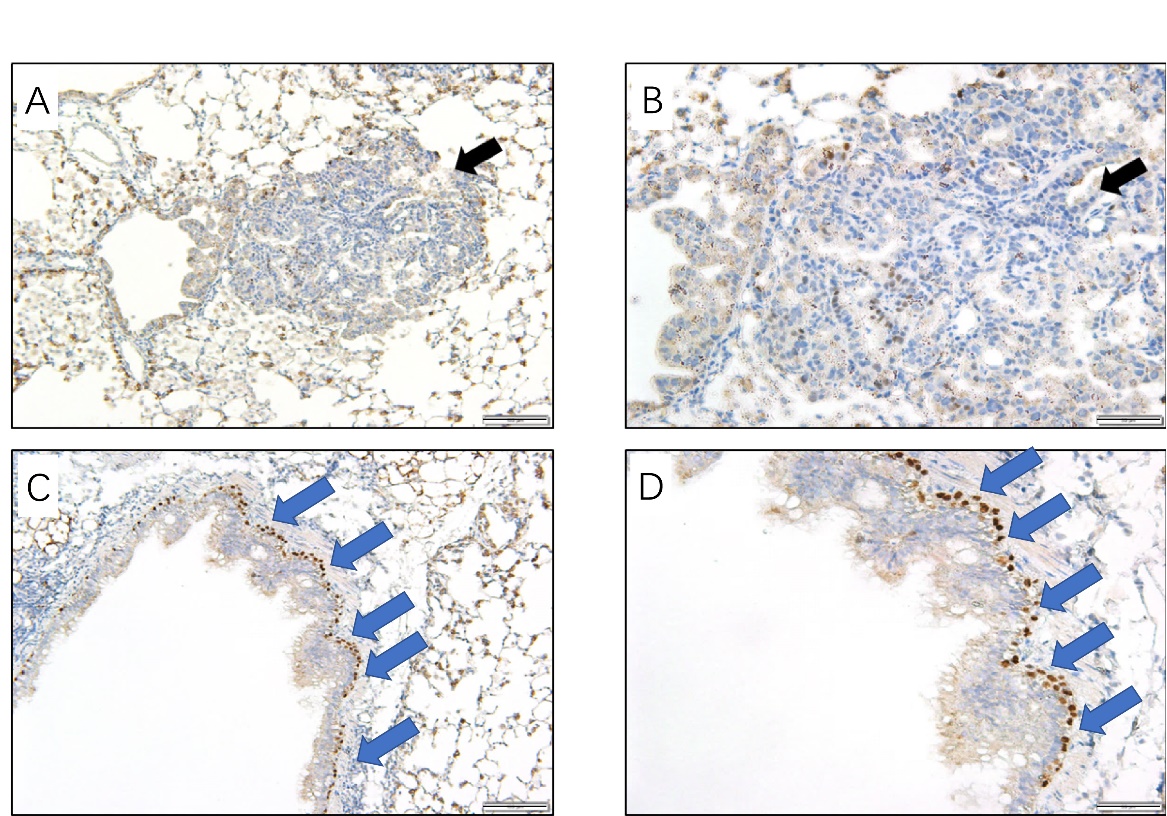


**Supplemental Figure 2 (Figure S2) IHC staining of P63 in lung tumors and normal lung epithelium of LSL-*ERK3/PTEN*^F/F^/CCSP-iCre mouse.** Prominent P63 staining is observed in normal epithelium (**C** and **D**) but not in tumors (**A** and **B**) of the same lung tissue section of LSL-*ERK3/PTEN*^F/F^/CCSP-Cre mice (n=6). **A** and **C:** 20 X magnification. **B** and **D**: 40 X magnification.
